# Cold ischemia-induced autophagy in rat lung tissue

**DOI:** 10.3892/mmr.2014.2999

**Published:** 2014-11-26

**Authors:** XU CHEN, JING-XIANG WU, XING-JI YOU, HONG-WEI ZHU, JIONG-LIN WEI, MEI-YING XU

**Affiliations:** 1 Department of Anesthesiology, Shanghai Chest Hospital, Shanghai Jiaotong University, Shanghai 200030, P.R. China; 2 Department of Physiology, Second Military Medical University, Shanghai 200433, P.R. China; 3 Department of Anesthesiology, Shanghai Huadong Hospital, Shanghai Fudan University, Shanghai 200002, P.R. China

**Keywords:** lung transplantation, cold ischemia preservation, autophagy

## Abstract

Autophagy is a highly conserved pathway that permits recycling of nutrients within the cell and is rapidly upregulated during starvation or cell stress. Autophagy has been implicated in the pathophysiological process of warm ischemia-reperfusion injury in the rat lung. Cold ischemia (CI) preservation for lung transplantation also exhibits cell stress and nutrient deprivation, however, little is known with regard to the involvement of autophagy in this process. In the present study, CI preservation-induced autophagy and apoptosis was investigated in the lungs of Sprague Dawley rats. Sprague Dawley rat lungs were flushed and preserved at 4°C (i.e. CI) for various durations (0, 3, 6, 12 and 24 h). The levels of autophagy, autophagic cell death and apoptosis were measured at each time point following CI. The results revealed that autophagy was induced by CI preservation, which was initiated at 3 h, peaked at 6 h after CI and declined thereafter. Additionally, a coexistence of autophagic cell death and apoptosis was observed in rat lung tissues following prolonged CI. These findings demonstrate that autophagy is involved in the pathophysiological process of lung CI. Furthermore, autophagic cell death in addition to necrosis and apoptosis occurs following CI in the lung. CI preservation may therefore be a potential mechanism of lung injury during organ preservation prior to lung transplantation.

## Introduction

Lung transplantation (LT) is the mainstay of therapeutic modalities for end-stage lung diseases, however, the 1-year survival rate following lung transplantation is only 80%, with primary graft dysfunction (PGD) contributing to 30% of mortalities (1). Ischemia-reperfusion injury (I/R) has been reported to be one of the main causes of PGD, therefore significant efforts have been made to optimize the methods for lung preservation in order to decrease the risk of lung injury during the period of ischemia (2,3). Several studies have suggested that prolonged durations of lung cold ischemia (CI), in combination with other donor characteristics, has a negative effect on the outcome following LT (1,4).

Autophagy is a cellular process in which autophagosomes deliver cytoplasmic proteins and macromolecules to lysosomes for degradation (5,6), and has been implicated in a number of diseases (7,8). Autophagy can lead to non-apoptotic programmed cell death, which is termed autophagic cell death (9,10). Increased autophagic activity and autophagic cell death have been identified in the damaged tissues of various disease models (11–14). A previous study demonstrated that autophagy is also involved in warm I/R injury in the lung (15).

Autophagy is often rapidly upregulated during starvation or cell stress (5,6). Since CI preservation for LT magnifies cell stress and nutrient deprivation, measuring the autophagic activity during CI would yield significant results. In addition to apoptosis, autophagic cell death has been hypothesized to be another important potential mechanism of neural tissue damage, induced by ischemia (13,14). However, whether autophagy is induced during CI of lung preservation and whether autophagic cell death contributes to lung tissue damage following CI remains to be elucidated. The present study verified that autophagy is involved in CI of lung preservation and autophagic cell death was observed in rat donor lungs following prolonged CI.

## Materials and methods

### Ethics

Ethical approval for the present study was provided by the Ethical Committee (no. KS1218) of the Shanghai Chest Hospital, affiliated to Shanghai Jiaotong University (Shanghai, China), on 10 July 2011.

### CI preservation

The present study was performed in accordance with the Guidelines for Animal Experiments of the Shanghai Jiaotong University (Shanghai, China). Male Sprague-Dawley rats weighing 250–300 g were housed with free access to food and water under a natural day/night cycle. All rats were acclimated for a week prior to any experimental procedures. The rats were anesthetized by an intraperitoneal injection of ketamine chloride (100 mg/kg; Jiangsu Hengrui Medicine Co., Ltd., Lianyungang, China) and intubated through a tracheotomy. Rats were mechanically ventilated with 100% oxygen, at a tidal volume of 10 ml/kg and a respiratory rate of 70 breaths/min. Following heparinization (1000 U/kg intravenously), a median sternotomy was performed. The pulmonary artery (PA) was cannulated through a right ventriculotomy and the left atrium was sheared directly. The lungs were flushed through the PA cannula with 20 ml of cold low-potassium dextran solution (Perfadex, Vitrolife, Gothenburg, Sweden) via gravity drainage of 25 cm H_2_O. The PA, vein and trachea were ligated and subsequently the heart-lung bloc was excised from the chest. Following the excision, the inflated lung was placed into low-potassium dextran solution and stored at 4°C for CI preservation. A total of 30 rats were randomly divided into five treatment groups and underwent en bloc heart-lung harvest as described previously **(**16**)**. These blocs were subsequently followed by CI preservation for varying time periods (0, 3, 6, 12 and 24 h, respectively). Following CI preservation, the left lung tissues of each bloc were fixed in 4% paraformaldehyde (Guangfu Fine Chemical Research Institute, Tianjin, China) in 0.01 M phosphate-buffered saline (PBS; Basic Medicine Faculty of Shanghai Jiaotong University, Shanghai, China) pH 7.4 at 4°C for 24 h. Subsequently, the samples were cryoprotected overnight in 30% sucrose in 0.01 M PBS at 4°C and embedded in optimal cutting temperature (OCT) media prior to immunofluorescence staining. The right lung tissues were frozen in liquid nitrogen and stored at −80°C prior to western blot analysis and reverse transcription quantitative polymerase chain reaction (RT-qPCR) analysis.

### Double immunofluorescence staining

Immunofluorescence staining was performed using transverse sections to investigate alterations in the expression of a specific autophagy marker, microtubule-associated protein 1 light chain 3B (LC3B), at each time point following CI. To identify DNA fragmentation (a marker of apoptosis) in the cells expressing LC3B, double staining of LC3B and the terminal deoxynucleotidyl transferase-mediated dUTP nick end labeling (TUNEL) were performed. Tissue sections (10 μm thickness) were prepared from OCT media-embedded tissue using a cryostat. The sections were washed in 0.01 M PBS and permeabilized in 0.2% Triton X-100 (Sigma-Aldrich, St. Louis, MO, USA). For immunofluorescent labeling, sections were pre-incubated in 20% goat serum (Beyotime Institute of Biotechnology, Nantong, China) for 30 min and subsequently incubated overnight at 4°C with the primary antibody, (LC3B, D11, XP^®^ rabbit monoclonal antibody used at 1:200; #3868s; Cell Signaling Technology, Inc., Danvers, MA, USA) in 0.01 M PBS. Following three washes in PBS, the sections were incubated for 2 h with Alexa Fluor 488-conjugated anti-rabbit immunoglobulin G antibody (1:500; A11008; Invitrogen Life Technologies, Carlsbad, CA, USA) in 0.01 M PBS at room temperature in the dark. TUNEL staining was applied using an *in situ* Cell Death Detection kit, TMR red (Roche Diagnostics GmbH, Mannheim, Germany), according to the manufacturer’s instructions. 4′,6-Diamidino-2-phenylindole (Beyotime Institute of Biotechnology) was employed for nuclear staining. Following rinsing in PBS, the sections were mounted with antifade solution (Beyotime Institute of Biotechnology). The immunoreactive signals were sequentially visualized in the same section using an LSM 710 Meta confocal microscope (Zeiss, Jena, Germany) with three distinct filters (405, 488 and 543 nm). The signal intensities through each filter were matched at the time of imaging. The images through each filter were individually optimized for brightness and background prior to generating the final composite images.

### LC3B-positive cell counting

The LC3B-positive cells, which were defined as the cells exhibiting punctuate LC3B fluorescent dots (14,17), were counted in the transverse sections scanned previously. A total of six serial sections were used for each sample. The LC3B-positive cells were counted in five random microscopic fields (magnification, ×40) of each section by an examiner who was blinded to the experimental conditions.

### Western blot analysis

Rat lung tissues were homogenized in cold T-PER™ lysis buffer (Pierce Biotechnology, Inc., Rockford, IL, USA) with a protease inhibitor cocktail (Roche Diagnostics GmbH). Subsequently, the lysates were quickly sonicated three times for 15 sec in an ice bath, boiled for 5 min at 95°C and stored at −80°C prior to use. Aliquots of proteins were separated by 10% sodium dodecyl sulfate polyacrylamide gel, transferred onto a polyvinylidene difluoride membrane and detected using antibodies against LC3B(D11) XP^®^ rabbit monoclonal antibody (1:1,000; #3868s; Cell Signaling Technology, Inc.), Beclin-1 (H-300) rabbit polyclonal antibody (1:1,000; sc-11427; Santa Cruz Biotechnology, Inc., Santa Cruz, CA, USA) or caspase-3 rabbit polyclonal antibody (1:1,000; #9662s; Cell Signaling Technology, Inc.), respectively. β-actin (1:5,000; Sigma Aldrich, Shanghai, China) was used as a loading control.

### RT-qPCR analysis

RT-qPCR was performed to assess autophagy-related gene 5 (Atg5), B-cell lymphoma 2 (Bcl-2) and Bcl-2-associated X protein (Bax) gene expression in lung samples. Total RNA was isolated using TRIzol (Invitrogen Life Technologies) according to the manufacturer’s instructions. Reverse transcription was completed using a PrimeScript 1st Strand cDNA Synthesis kit (Takara Biotechnology Co., Dalian, China) according to the manufacturer’s instructions. SYBR^®^ Green qPCR was performed using an Applied Biosystems 7500 real-time PCR System (Applied Biosystems, Foster City, CA, USA) in a 20 μl reaction containing 2 μl of cDNA, 10 μl of 2X SYBR^®^ Premix Ex Taq™ (DRR041A, Takara Biotechnology Co.) and primers designed specifically for the genes amplified (18). The primer sequences are shown in Table I (Sangon Biotech, Shanghai, China).

### Statistical analysis

Data are expressed as the mean ± standard deviation. The statistical significance was estimated by one-way analysis of variance followed by the Student-Newman-Keuls test. P<0.05 was considered to indicate a statistically significant difference.

## Results

### Autophagy is induced following CI

**Figure 1:**
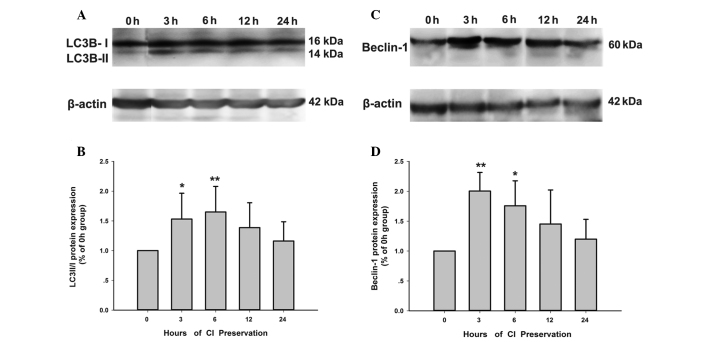
Protein expression of LC3B and Beclin-1 in lung tissues following varying time periods of CI preservation. (A) Representative immunoblots of LC3B. (B) Comparisons of LC3B-II/I ratio and Beclin-1. (C) Representative immunoblots of Beclin-1 protein expression in lung homogenates at different time points of CI preservation. (D) Comparisons of ratios of Beclin-1. Protein levels among different groups were determined from six independent experiments. The data represent the mean ± standard deviation of six separate experiments. ^*^P<0.05, ^**^P<0.01 vs. 0 h group. CI, cold ischemia; LC3B, microtubule-associated protein 1 light chain 3B.

To assess autophagic activity in lung tissues following CI, the protein expression of LC3B-I and LC3B-II (autophagosome marker proteins) was examined using western blot analysis. A significant increase in the ratio of LC3B-II/I was observed after 3 h of CI, peaked at 6 h and subsequently decreased to the basal level after 12 h (Fig. 1A and B). This result suggested that autophagic activity was induced in lung tissues following CI. Consistently, changes in the level of the protein Beclin-1, another key autophagic protein, exhibited a similar trend (Fig. 1C and D).

### Immunofluorescence staining of LC3B-positive cells

**Figure 2:**
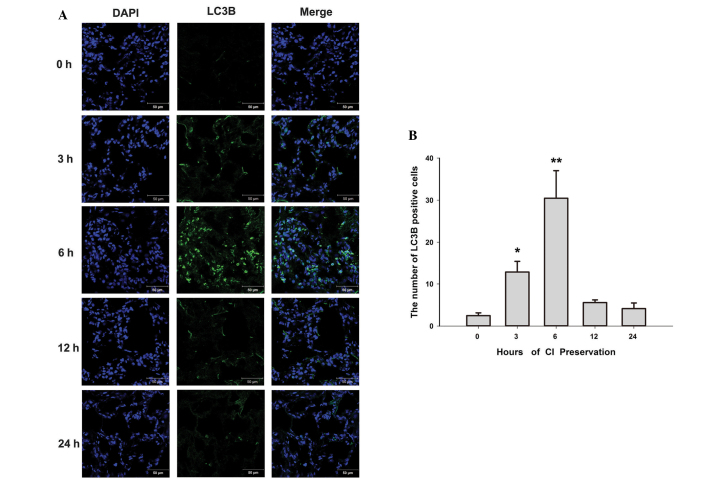
Number of LC3B-positive cells in transverse sections at different time points of CI preservation. (A) Representative immunofluorescence staining of LC3B. Scale bar=50 μm. (B) Number of LC3B-positive cells was compared among different time points of CI preservation. The data represent the means ± standard deviation of 30 transverse sections per time point. ^*^P<0.05, ^**^P<0.01 vs. 0 h group. CI, cold ischemia; LC3B, microtubule-associated protein 1 light chain 3B.

**Figure 3:**
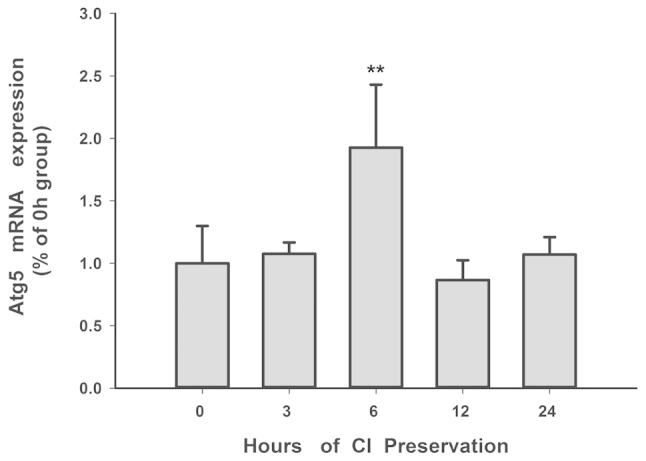
mRNA expression of *Atg5* in lung tissues during CI preservation. The mRNA level of *Atg5* was measured by reverse transcription quantitative polymerase chain reaction. The data, expressed as a percentage of the 0 h group, represent the mean ± standard deviation of three separate experiments. ^**^P<0.01 vs. 0 h group (one-way analysis of variance followed by Student-Newmans-Keuls test). ATG5, autophagy protein 5; CI, cold-ischemia.

In order to confirm these findings, the LC3B-positive cells were detected by immunofluorescence staining. A marked increase in the number of LC3B puncta was observed in lung tissue within the prolonged CI time, which is consistent with the trend observed in western blot analysis. The marked increase in LC3B-positive cells was initiated at 3 h, peaked at 6 h following CI and thereafter decreased to the basal level after 12 h of CI preservation (Fig. 2). In order to further confirm autophagy induction in lung tissues following CI, the mRNA level of another important autophagy marker, *Atg5*, was assessed via qPCR analysis. In a similar manner to that of LC3B induction, a significant increase in *Atg5* expression was detected in the 6 h group compared with the 0 h group following CI (Fig. 3).

### Autophagic cell death and apoptosis in lung tissues following CI

**Figure 4:**
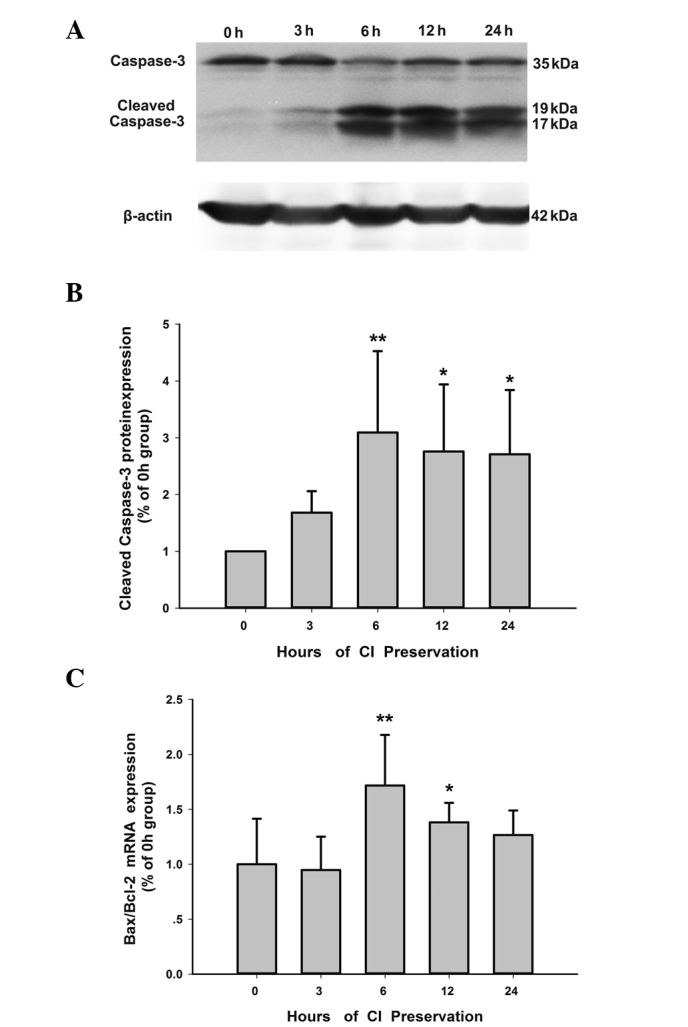
Changes in caspase-3 protein level and Bax/Bcl-2 mRNA ratio in lung tissues during CI preservation. (A) Representative immunoblots of caspase-3 in lung tissues at different time points of CI preservation. (B) Protein levels of cleaved caspase-3 at different time points were analyzed. The data, expressed as a percentage of the 0 h group following the adjustment of β-actin (a loading control), represent the means ± standard deviation of three separate experiments. (C) Ratio of Bax/Bcl-2 mRNA expression following different time periods of CI preservation. ^**^P<0.01 vs. 0 h group. CI, cold-ischemia; Bcl-2, B-cell lymphoma 2; Bax, Bcl-2-associated X protein.

**Figure 5:**
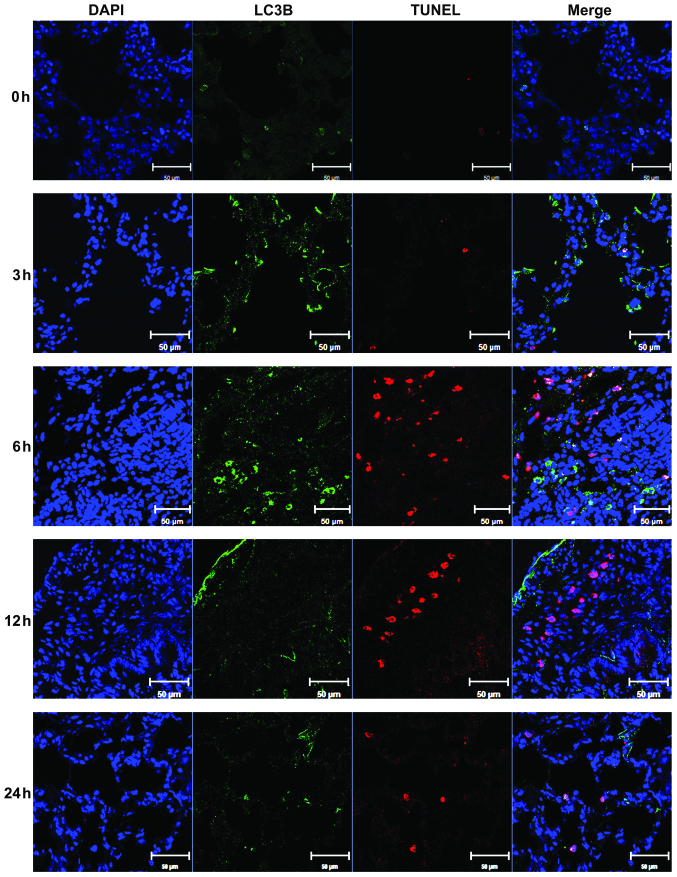
Double-immunofluorescence analysis of LC3B and TUNEL in lung tissues following CI preservation. Transverse sections prepared from lung tissues following different time periods of CI preservation were subjected to LC3B immunofluorescent staining and TUNEL assay. 4′,6-Diamidino-2-phenylindole was used to stain the cell nuclei. Compared with the 0 h group, the number of TUNEL-positive cells and LC3B-positive cells evidently increased after 6 h of CI preservation. Scale bar=50 μm. CI, cold ischemia; LC3B, microtubule-associated protein 1 light chain 3B; TUNEL, terminal deoxynucleotidyl transferase-mediated dUTP nick end labeling.

**Figure 6:**
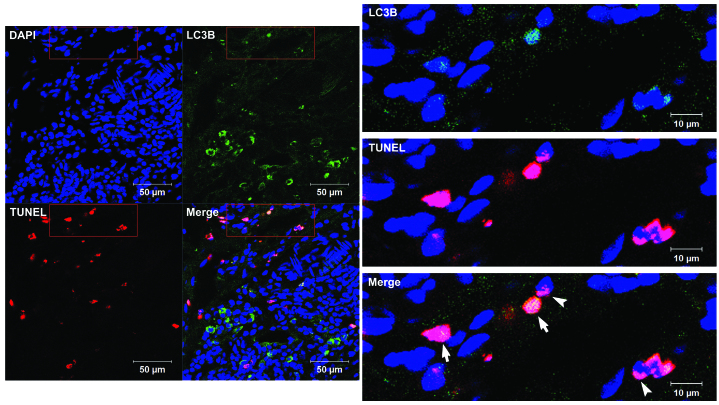
Autophagic cell death in lung tissues after 6 h of CI preservation. The LC3B-positive cells were observed in TUNEL-positive cells. The nuclei in TUNEL- and LC3B-positive cells were round, which was consistent with autophagic cell death (arrow). The shrunken or fragmented DNA was observed only in apoptotic nuclei (arrowheads). Scale bar=50 μm in the upper panel images and 10 μm in the lower panel images. CI, cold ischemia; LC3B, microtubule-associated protein 1 light chain 3B; TUNEL, terminal deoxynucleotidyl transferase-mediated dUTP nick end labeling.

Autophagic cell death and apoptosis in lung tissues following CI was also determined. Apoptosis in lung tissues following CI was initially verified through analyzing the changes in the expression level of an apoptosis marker enzyme, caspase-3, in each group. The level of cleaved caspase-3, the active form, markedly increased between 6 and 24 h post-CI compared with the basal level at 0 h (Fig. 4A and B). In addition, the ratio of mRNA of the pro-/anti-apoptotic genes Bax/Bcl-2 significantly increased between 6–12 h of CI preservation (Fig. 4C). The present results verified marked apoptosis in the lung CI model. To identify autophagic cell death and apoptosis in lung tissues following CI, double-immunofluorescence analysis of LC3B and TUNEL (an apoptosis-specific staining assay) was performed. The results clearly demonstrated that the levels of TUNEL-positive cells and the LC3B-positive cells increased at 6 h after CI, compared with the 0 h group (Fig. 5). The LC3B-positive signals only partially colocalized with the TUNEL signal when merged. Notably, the nuclei of the TUNEL-positive, LC3B-negative cells were shrunken or fragmented, indicating the presence of typical apoptotic nuclei. By contrast, the nuclei of TUNEL- and LC3B- double positive cells were round, as usually observed in autophagic cell death (19) (Fig. 6).

## Discussion

Although significant progress has been made in the study of the function of autophagy in a number of ischemic conditions *in vitro* and *in vivo*, little is known with regard to autophagy in lung tissues following CI preservation. The present study revealed that autophagy is involved in lung CI preservation and autophagic cell death in rat donor lungs that undergo prolonged CI. This process may be involved in CI-induced lung injury. These hypotheses are supported by the following experimental evidence: i) In a lung CI model, autophagy was induced and found to reach a peak at 6 h after CI compared with the basal level at 0 h; ii) following 12 h of CI, the autophagic markers decreased to the basal level. In addition to apoptosis, autophagic cell death was also induced by lung CI preservation, which was identified by double-immunofluorescence analysis of LC3B and TUNEL.

Autophagy is a tightly regulated and highly conserved physiological process involving the recycling of cytoplasmic proteins and molecules within autophagolysosomes (20,21). Starvation, hypoxemia and energy depletion are potent stimulators of autophagy, *in vitro* and *in vivo* (21). Under these conditions, autophagy is hypothesized to enable cell survival by recycling essential amino acids and cell metabolites (5,6,22). The preservation of donor organs involves the rapid cooling of *ex vivo* organs to 4°C (23). Although hypothermia is essential for the preservation of donor lungs and significantly extends organ viability, it is associated with inactivation of the sodium pump, oxidative stress and the release of proinflammatory mediators (1,24). Additionally, donor organs are subjected to nutrient deprivation and energy depletion. These events are effective triggers for autophagy, as has been demonstrated in the liver (25–28), kidney (29–31) and heart (32–35). The results in the present study demonstrated that the protein or mRNA levels of three autophagic markers (LC3B, Beclin-1 and *Atg5*) in rat lung tissues significantly increased after 3–6 h of CI preservation, indicating that autophagy was significantly induced in the rat lung tissues during CI preservation.

By contrast, autophagy has also been hypothesized to be involved in cell death (32,36–40). At present, at least three different types of cell death (necrosis, apoptosis and autophagic cell death) are considered to be involved in a number of ischemic events. Previous studies, however, have only focused on necrosis and apoptosis, but not autophagic cell death, as potential mechanisms of lung damage following CI preservation (41,42). Autophagic cell death can be morphologically differentiated from apoptotic cell death. The nucleus is shrunken and fragmented in apoptosis, while it remains round in autophagic cell death (14,19,43). In the present study, immunostaining results demonstrated a partial colocalization of LC3B and TUNEL signal in rat lung tissues following CI preservation. The nuclei of TUNEL-positive and LC3B-negative cells were shrunken or fragmented, which were typical apoptotic nuclei; however the nuclei of the cells positive for TUNEL and LC3B were observed to be round, which was consistent with the nuclear morphology of autophagic cell death. These results suggest that autophagic cell death may be involved in rat lung injury following CI preservation.

There remains a number of limitations to the present study. For example, to what extent the final pulmonary function may be affected by autophagy and autophagic cell death during prolonged CI preservation remains to be elucidated. Further investigations are required to elucidate whether the function of autophagy is protective or detrimental for donor lung tissue during CI and by which molecular mechanism autophagy was induced in CI preservation. In addition, the association between autophagy and apoptosis during CI preservation also requires detailed investigation.

In conclusion, to the best of our knowledge this is the first study demonstrating the activation of autophagy and induction of autophagic cell death in lung tissue following CI preservation. The present results suggest that CI preservation may induce cell death in rat lung tissues through apoptosis, as well as through autophagic cell death simultaneously, which may be considered an important factor in future therapeutic approaches to organ preservation for lung transplantation.

## Figures and Tables

**Table I:** Polymerase chain reaction primers.

Genes	Forward 5′-3′	Reverse 5′-3′
*Atg5*	AGTGGAGGCAACAGAACC	GACACGAACTGGCACATT
*Bcl-2*	GGATGACTTCTCTCGTCGCTAC	TGCAGATGCCGGTTCAG
*Bax*	GCAGGGAGGATGGCTGGGGAGA	TCCAGACAAGCAGCCGCTCACG
*β-actin*	GTCAGGTCATCACTATCGGCAAT	AGAGGTCTTTACGGATGTCAACGT
